# Controversies about a common etiology for eating and mood disorders

**DOI:** 10.3389/fpsyg.2014.01205

**Published:** 2014-10-27

**Authors:** Clara Rossetti, Olivier Halfon, Benjamin Boutrel

**Affiliations:** ^1^Center for Psychiatric Neuroscience, Lausanne University HospitalLausanne, Switzerland; ^2^Division of Child and Adolescent Psychiatry, Department of Psychiatry, Lausanne University HospitalLausanne, Switzerland

**Keywords:** depression, obesity, reward, overweight, palatable food

## Abstract

Obesity and depression represent a growing health concern worldwide. For many years, basic science and medicine have considered obesity as a metabolic illness, while depression was classified a psychiatric disorder. Despite accumulating evidence suggesting that obesity and depression may share commonalities, the causal link between eating and mood disorders remains to be fully understood. This etiology is highly complex, consisting of multiple environmental and genetic risk factors that interact with each other. In this review, we sought to summarize the preclinical and clinical evidence supporting a common etiology for eating and mood disorders, with a particular emphasis on signaling pathways involved in the maintenance of energy balance and mood stability, among which orexigenic and anorexigenic neuropeptides, metabolic factors, stress responsive hormones, cytokines, and neurotrophic factors.

## INTRODUCTION

Most of antidepressant medications have long been known to induce weight gain, although the obesogenic mechanisms of these treatments remain largely unknown. Most interestingly, a large body of evidence supports the idea that non medicated depressed patients with mood disorders have increased risks of developing obesity compared to the general population, while specific interventions aiming at reducing body weight (including bariatric surgery and increased physical activity) have been found to improve mood. Hence, empirical observations have long suggested a common etiology between obesity and depression: (1) unhealthy diets favoring energy dense foods promote the development of both pathologies; (2) reduced physical activity and sedentary lifestyle are commonly observed in obese and depressed patients; (3) impaired sleep and/or circadian cycles deteriorate mood and increase body weight; (4) recurrent psychological stress and early life trauma have been shown to contribute to a late-onset obesity and depression. Considering the increased prevalence of obesity and depression worldwide, a better comprehension of their common biological bases has become crucial for identifying novel therapeutic targets.

Indeed, obesity-related diseases, including type II diabetes, high blood pressure, and cancer, have become one of the leading causes of preventable death worldwide, reducing life expectancy by 10 years or so. With depression, obesity represents a serious public health concern that contributes to significantly deteriorate the quality of life in people of most countries. Although researches concerning obesity and depression have been conducted for decades separately, recent evidence has shown that these two pathologies might share some neurobiological underpinnings, and possibly a common etiology. Here, we propose to review biological adaptations within several signaling pathways potentially underlying the increased risks of developing both diseases.

Obesity is a pathological condition characterized by excessive body fat accumulation resulting from an imbalance between caloric intake and expenditure. The World Health Organization (WHO) has regularly monitored the worldwide prevalence of overweight and obesity, and a recent estimation suggests that 11% of the world population (more than half a billion people) is obese, and 35% overweighed. Some monogenic forms of obesity have been described (e.g., mutations of leptin and leptin receptor, pro-opiomelanocortin and melanocortin-4-receptor genes) but they are very rare and not sufficient to explain the worldwide distribution of this disorder (Andreasen and Andersen, 2009). As a matter of fact, recent clinical findings rather suggest that obesity is most likely related to common genetic variants and/or single-nucleotide polymorphisms, representing a genetic risk factor of vulnerability (Frayling et al., 2007; Andreasen et al., 2008; Haupt et al., 2008; Hunt et al., 2008).

Depression is the commonest psychiatric disorder, characterized by severe negative mood and inability to experience pleasure from usually pleasurable activities (anhedonia). Depressed mood is associated with fatigue, incapacity to concentrate, altered appetite, sleep disorders, and metabolic complications (Ustun et al., 2004; Kessler and Bromet, 2013). Depressive symptoms often occur at younger age and, in the most serious cases, lead to suicide attempts. The most recent survey conducted by WHO in 2012 estimates that depression affects more than 350 million people worldwide, hence constituting a major threat to public health (Kessler and Bromet, 2013). Major depression is a heterogeneous disease comprising several subtypes, but the atypical depression is one of the most diagnosed forms. Symptoms like psychomotor retardation, insomnia, increased appetite, and body weight gain differentiate atypical depression from the other forms (Gold and Chrousos, 2002). This subtype of depression is often accompanied by visceral adiposity and shares with obesity certain metabolic, endocrine and behavioral alterations (Vogelzangs et al., 2007; Lasserre et al., 2014). Interestingly, fat accumulation, commonly measured by the body mass index (BMI; weight in kilograms divided by height in squared meters, BMI), has long been used to monitor weight gain. Increasing evidence rather suggests that abdominal obesity [as referred as the waist-to-hip ratio (WHR)], presents a stronger predictive value than total body fat for predicting obesity-depression co-morbidity (Zhao et al., 2011; Wiltink et al., 2013).

Like obesity, the underlying biological causes of depression remain largely unknown, but genetic predisposition is considered to account for ∼40% of cases (Uhl and Grow, 2004). The genetic approach suggests that depression is a polygenic disease, determined by environmental influences in genetically predisposed individuals (Charney and Manji, 2004). Early life stress has been considered as one of the most critical factor triggering depression (Nemeroff and Vale, 2005). Many longitudinal studies have shown that depression is a consistent predictor of metabolic syndrome since its progression is often associated with cardiovascular diseases, diabetes, obesity, and chronic inflammation (Katon and Ciechanowski, 2002). One possible explanation for this observation would take into account the fact that depressed patients often adopt poor health behaviors, including smoking and drinking habits (Davis et al., 2008), reduced physical activity and unhealthy diets (Cizza, 2011).

The aim of this review is to summarize the current knowledge about the obesity-depression relationship and discuss plausible biological mechanisms underlying this association; first by reviewing the clinical findings supporting the intermingled interaction between obesity and depression, second by discussing the possibility that obesity may be a cause of depression and *vice versa*. In particular, we will be focusing our attention on signaling pathways involved in the maintenance of energy balance and mood stability, among which orexigenic and anorexigenic neuropeptides, metabolic factors, stress responsive hormones, cytokines, and neurotrophic factors.

## THE OBESITY-DEPRESSION ASSOCIATION AND MOST RELEVANT MEDIATORS

Since obesity has long been considered a metabolic illness, while depression was classified a psychiatric disorder, both basic science and medicine have long investigated these two pathologies separately. Most of the epidemiological and clinical investigations conducted before the end of the 20th century provided only confounding outcomes, unable to prove a direct association between obesity and depression. Nevertheless, a seminal observation proposed that “socio-demographic, psychosocial and genetic factors may render certain obese individuals more prone to depression and *vice versa*” (Faith et al., 2002). The overlap between mood disorders and obesity was later supported by an extensive study reviewing clinical studies from 1966 to 2003 (McElroy et al., 2004). The authors claimed that obesity and mood disorders were likely related disorders with a distinct, but overlapping, pathophysiology. As a consequence, only some forms of obesity and mood disorders that share pathogenic factors would be related. Further supporting this assumption, obesity was found to be strongly associated with a range of common mood and anxiety disorders, suggesting that social and cultural factors may represent possible mediators and/or modulators of the obesity-depression relationship (Simon et al., 2006). More recent publications, including prospective cohort studies and cross-sectional researches, also reported relevant observations supporting a bidirectional causal link between obesity and depression, by which each disease may contribute to the other (Markowitz et al., 2008; Luppino et al., 2010; Faith et al., 2011). In a recent systematic review of the literature, undertaken to identify biopsychological variables associated with the relationship between obesity and depression (Preiss et al., 2013), physical health, decreased social activity, family depression history, childhood abuse, body image distorsion, severity of obesity and binge eating were listed consistent risk factors for developing comorbid obesity and depression. Collectively, these reports suggest that specific risk factors may confer to subgroups of obese individuals higher probability to develop depression, and to subgroups of depressed patients greater vulnerability to become obese. However, it is important to note that the conclusions drawn from of Lupino’s, Faith’s, and Preiss’s works may be limited by quite a strong heterogeneity, in the methodology, the patient identification and the measures of depression and body weight. In particular, the evidence for a mutual influence of depression and obesity relied on reported weight and not on objective body weight measures (Faith et al., 2011), and the negative impact of obesity on depression depended on the evaluation of clinical depression instead of depressive symptoms (Luppino et al., 2010).

Hence, despite limitations, current data suggest a mutual influence between obesity and depression (Atlantis and Baker, 2008), even if a few studies on adolescents would argue that depression represent a clearer risk factor for obesity (Korczak et al., 2013), whereas obesity may not confer any higher risk for later developing depressive symptoms (Roberts and Duong, 2013). A reasonable conclusion would imply that obesity might represent a risk factor for depression only under particular conditions, which are binge eating behavior or abdominal fat deposition (Weber-Hamann et al., 2002; Araujo et al., 2010; Zhao et al., 2011; van Reedt Dortland et al., 2013a,b).

To summarize, the obesity-depression association seems to be strongly dependent on several risk factors that have been clearly established. Reduced physical activity and sedentary lifestyle have been frequently observed in depressed (Azevedo Da Silva et al., 2012; Song et al., 2012) and obese individuals (Bailey et al., 2007; Tucker and Tucker, 2011). Conversely, when depressed patients were encouraged to do physical exercise, improvement of their mood has been repeatedly reported (Conn, 2010; Carek et al., 2011; Rimer et al., 2012). Moreover, the efficacy of exercise in reducing risk of depression has also been observed in overweight and obese adults (Vallance et al., 2011). Sleep disorders and circadian cycle alterations have been shown to be associated with mood changes (Costa e Silva, 2006; Turek, 2007; Kronfeld-Schor and Einat, 2012; Avila Moraes et al., 2013) and weight gain (Bray and Young, 2007; Shi et al., 2013). Gender has also been considered an important factor since a stronger vulnerability in women has been well documented for both diseases (Peveler et al., 2002). Unhealthy diets characterized by excessive consumption of energy dense foods have been associated with an increased risk of depression (Jeffery et al., 2009; Sanchez-Villegas et al., 2012; Sanchez-Villegas and Martinez-Gonzalez, 2013) as well as obesity (Fulton, 2010; Mozaffarian et al., 2011). In contrast, adherence to Mediterranean diet or to others diets comprising high amounts of vegetables and fruits have been shown to reduce risks of depression (Sanchez-Villegas et al., 2009; Jacka et al., 2010). Finally, stress and early life trauma have been shown to significantly contribute to both late-onset obesity (Gustafson and Sarwer, 2004; Gunstad et al., 2006; D’Argenio et al., 2009) and depression (Cirulli et al., 2009).

In the next part of this article, we will be essentially focusing on two increasing risk factors: stress and diet.

## OBESITY AS A CAUSE OF DEPRESSION

The decision to eat is not only ultimately influenced by the internal state of the caloric equation but also by non-homeostatic factors, including food palatability and environmental cues known to trigger conditioned responses (Lutter and Nestler, 2009; Williams and Elmquist, 2012). Hence, a current consensus acknowledges that feeding behaviors are not only influenced by caloric needs, but also by other layers of regulation that involves the processing of reward, notably through dopamine signaling and its ability to pair food consumption to the context predicting its availability (see for review, Volkow et al., 2013a). In an evolutionary perspective, this property of palatable foods used to be critical for surviving in environments where food sources were scarce. Large amounts of food were eaten when available, enabling energy to be stored in the body (as fat) for future use (Spiegelman and Flier, 2001). However, food habits have profoundly changed over the past decades. Energy-dense foods, especially high fat diets, have a reduced satiety capacity and a higher hedonic value compared to that of meals richer in proteins and/or complex carbohydrates, which may explain their excessive consumption and their role in promoting overweight and obesity (Lawton et al., 1993; Blundell and Macdiarmid, 1997). Moreover, in modern societies, food has become plentiful and ubiquitous. As a consequence, the evolutionary adaptation inducing energy storing has become a dangerous liability (Erlanson-Albertsson, 2005a,b) promoting disinhibited and uncontrolled food seeking habits. Energy dense foods are potentially harmful for human health not only for their unbalance contents but also for their capacity to promote overeating behaviors (Berthoud et al., 2011; Egecioglu et al., 2011).

Recent evidence has established that disruption of energy homeostasis can affect the reward circuitry and that overconsumption of palatable food can lead to changes in the reward circuitry that result in compulsive food intake (Bassareo and Di Chiara, 1999; Kenny, 2011). The peripheral signals include peptides and hormones like leptin, insulin, cholecystokinin (CCK), tumor necrosis factor- α (TNF-α) but also nutrients (sugars and lipids), that are transported via the vagus nerve to the nucleus solitary tract and directly through receptors located not only in the hypothalamus, but also in autonomic and limbic brain regions. These multiple signaling pathways ensure that food is consumed when needed. However, with repeated access to highly palatable food, some individuals may eventually override the inhibitory processes that signal satiety and begin to compulsively consume large amounts of food despite nutrition overload (Zheng and Berthoud, 2007; Johnson, 2013). This loss of control and compulsive pattern of food intake is reminiscent of the drug intake patterns seen in addiction and has led to the description of some types of eating disorders inducing obesity as a form of “food addiction” (Volkow et al., 2013b).

Smith and Robbins proposed that overconsumption of palatable foods would lead to habit-driven responses by initiating a devolution from goal-directed to habitual behavior. According to this hypothesis, a process of signaling transfer would devolve impulsively driven and hedonically motivated actions from ventral to dorsal striatal control (Smith and Robbins, 2013). As a consequence, the consumption of high-fat/high-sugar foods would become less pleasurable and instead would turn into a compulsive response triggered by cues such as advertisements, mood, and context. The prefrontal cortex is considered to be critical for self-control, inhibition, and goal representation, and reduced activity in this region is associated with higher levels of impulsivity and compulsivity. Since executive function difficulties have been reported in overweight and obese individuals, and a decrease in orbito-frontal cortex volume correlated with disinhibited eating in obese adolescents, the shift from ventral to dorsal striatal control described above may also be associated with impairments in executive functions such as cognitive control, flexibility, decision-making, and working memory. Ultimately, individuals who experience a lack of control in the face of food persistently overuse their preferred food despite severe health, social, legal, and financial problems; and are unsuccessful at attempting to cut back or reduce their consumption. These behaviors are typically accompanied by feelings of guilt, remorse, sometimes triggering excessive food restriction and distress that in turn, promote palatable food intake to alleviate these dysphoric signs, further accentuating the spiraling distress (Fulton, 2010).

In other words, negative mood, provoked by palatable food withdrawal or by the incapacity to lose weight, often results in the adoption of an abnormal eating behavior characterized by recurrent cycles of dieting and overeating (Polivy and Herman, 1985; Petroni et al., 2007; Stice et al., 2008). Consequently, overeating episodes may turn into an overt binge-eating disorder (BED) that is frequently associated with a feeling of distress. Consistent with this, obese patients suffering from BED often present severe depressive symptoms (Heatherton and Baumeister, 1991; Stice et al., 2000; Spoor et al., 2006; Araujo et al., 2010; Blumenthal and Gold, 2010; Faulconbridge and Bechtel, 2014). These clinical findings are largely supported by experiments in which compulsive overeating has been induced in laboratory animals. For example, the capacity of palatable foods, when taken in a discontinuous pattern, to promote binge eating behavior has been repeatedly demonstrated in rodents (Colantuoni et al., 2002; Bello et al., 2003; Cottone et al., 2008; Corwin et al., 2011). Interestingly, several of these works have also proved that long-term intermittent access to palatable food is accompanied by depressive-like phenotypes and metabolic alterations (Cottone et al., 2009b; Rossetti et al., 2013).

Traumatic stress experiences, especially in early life, are known to affect mood and feeding behavior and therefore, they constitute a strong element conferring higher vulnerability for developing obesity and depression. Compelling evidence suggests that stress increases palatable food preference and consumption both in humans (Gluck et al., 2004; Gluck, 2006; Adam and Epel, 2007; O’Connor et al., 2008; Tryon et al., 2013a,b) and in rodents (Foster et al., 2006; Moles et al., 2006; Machado et al., 2013; Patterson and Abizaid, 2013). A likely explanation for such stress-induced preference is that palatable food acting as a “comfort food” would be able to alleviate discomfort (Jeffery et al., 2009). In agreement with this hypothesis, a large body of preclinical studies has demonstrated that, not only chronic stress promotes palatable food intake (Dallman et al., 2003; Pecoraro et al., 2004) but its withdrawal increases stress sensitivity and depressive-like behaviors (Avena et al., 2008; Teegarden and Bale, 2008; Iemolo et al., 2012).

Industrial foods that progressively replace healthier fresh cooked meals worldwide are harmful for human health not only for their strong rewarding properties but also for their low content of poly-unsatured fatty acids (PUFAs). Accordingly, higher intake of saturated fatty acids has been found to promote visceral fat deposition and mood alterations (Schulze et al., 2006; Akbaraly et al., 2009; Molenaar et al., 2009; Jacka et al., 2010). Diets containing low levels of PUFA also increase the risk of depression (Appleton et al., 2010), while lower levels of omega-3 PUFA have been recently reported in the blood of depressed patients (Ross et al., 2007; Lin et al., 2010). Similar conclusions about the role of PUFA in depression have been indirectly drawn from people adhering to a Mediterranean diet. This diet, privileging vegetables, fruits and fish and containing high levels of PUFA, has been shown to limit excessive weight gain and obesity (Schroder et al., 2004, 2007; Romaguera et al., 2009) as well as depression (Sanchez-Villegas et al., 2009). Beside these human studies, the relevance of PUFA in the prevention of depressions has also been reported in rodents (Fedorova and Salem, 2006; Huang et al., 2008) and non human primates (Chilton et al., 2011).

In sum, protracted consumption of unhealthy diets and recurrent stress could interfere with the homeostatic control of the energetic balance and modify the reactivity of the rewarding system leading ultimately to a loss of control over food intake. The resulting compulsive overeating might trigger a BED and eventually lead to abdominal fat deposition, two factors tightly associated with depression (Weber-Hamann et al., 2002; Rivenes et al., 2009; Zhao et al., 2011; van Reedt Dortland et al., 2013a,b).

### LEPTIN SIGNALING

Leptin is a peptide hormone belonging to the adipokines family that is secreted primarily from adipocytes of the white adipose tissue. Leptin serum levels change depending on the feeding/fasting state and correlate with body mass (Seeley and Woods, 2003). This peptide is coded by the obese gene *ob* and exerts its physiological function mainly in the brain. After release in the bloodstream, leptin pass across the blood–brain barrier (BBB) using a receptor-mediated transport (Banks et al., 1996). LepRb is the long form of the leptin receptor and is critical for leptin signaling cascade (Banks et al., 2000). LepRb was first identified in the hypothalamus and its activation was related to the anorectic effect of leptin, that is its ability to reduce food intake and increase energy expenditure (Chehab, 2000). Later, the presence of this receptor in other brain structures, namely the hippocampus, prefrontal cortex, ventral tegmental area (VTA) and amygdala has suggested additional roles of leptin in other physiological functions, among which learning and memory (Farr et al., 2006; Harvey et al., 2006), motivation for reward (Farooqi et al., 2007; Grosshans et al., 2012), and mood regulation (Guo et al., 2013; Milaneschi et al., 2014).

The role of leptin in the orchestration of food intake and energy expenditure in the hypothalamus has been largely described in the literature (Cone, 2005; Coll et al., 2007). Briefly, leptin is released by the adipose tissue, crosses the BBB and reaches the arcuate nucleus (ARC) of the hypothalamus where interacts with its receptors localized on two neuron populations: the orexigenic neuropeptide Y (NPY)/agouti related peptide (AgRP) neurons and the anorexigenic proopiomelanocortin (POMC)/cocaine and amphetamine regulated transcript (CART) neurons. Leptin inhibits the former and activates the latter, leading to a satiety signal (Spiegelman and Flier, 2001). It has long been known that impairment in leptin signaling causes obesity, increased food intake and fat deposition (Pelleymounter et al., 1995a; Speakman et al., 2007). These observations gave rise to the hypothesis that leptin could be used as anti-obesity treatment, but clinical investigations have shown that obese patients have chronically high levels of circulating leptin (Lu et al., 2006). This apparent contradiction suggests the emergence of a leptin resistance syndrome, a phenomenon also observed with insulin in diabetic patients. The precise mechanism responsible for leptin resistance is not yet known but may be linked to down-regulation of leptin receptors, reduced transport of leptin across the BBB or altered intracellular transduction of leptin signaling (Jung and Kim, 2013).

Abnormalities in leptin functioning are also believed to enhance the motivation for palatable food and promote its overconsumption. This assumption comes from the discovery that leptin receptors are also localized in brain reward structures (Figlewicz and Benoit, 2009). In particularly, leptin receptors have been detected in dopaminergic neurons of the VTA and electrophysiological studies in rodents have established that leptin decreases the firing rate of these neurons reducing dopamine release and food intake (Hommel et al., 2006). There is also evidence that leptin is able to regulate the incentive salience of reward because food restriction in rodents (that corresponds to a reduction of leptin signal) increases the preference for sucrose and other drugs in a conditioned place-preference paradigm (Figlewicz and Benoit, 2009). In other words, leptin impairment may result in a higher stimulation of dopaminergic neurons that, in turn, may increase the incentive for palatable food and ultimately its consumption. In support of this hypothesis, some animal studies demonstrated that injection of leptin in the VTA reduced food intake whereas a viral-mediated knockdown of leptin receptor in the same brain structure had opposite effect (Hommel et al., 2006).

In the last years, the scientific interest for leptin has also been extended to psychiatric disorders, such as anxiety and depression. Mutant mice that lack leptin signaling have been found to develop depressive symptoms (Collin et al., 2000; Sharma et al., 2010; Yamada et al., 2011) whereas systemic and central administration of leptin in wild-type mice reduced anxiety and depressive-like behaviors (Asakawa et al., 2003; Lu et al., 2006; Finger et al., 2010; Liu et al., 2010; Yamada et al., 2011; Guo et al., 2013). In addition, other preclinical studies have shown that circulating leptin levels are modulated by stress since chronic unpredictable stress or chronic social defeat, but not acute stress, decreased basal levels of leptin in rats (Lu et al., 2006; Ge et al., 2013).

Although these findings seem to support a reduction of leptin levels in animal models of depression, the current knowledge on the role of leptin signaling in human depression remains unclear. Leptin levels in depressed patients have been reported to be lower, higher or equal to those observed in control patients (Deuschle et al., 1996; Antonijevic et al., 1998; Rubin et al., 2002; Esel et al., 2005; Zeman et al., 2009). However, studies with larger size samples reported a negative correlation between plasma leptin levels and major depression, hence supporting the idea that decreased leptin signaling may be a shared biological alteration in both obesity and depression (Kraus et al., 2001; Atmaca et al., 2002; Westling et al., 2004; Jow et al., 2006; Lawson et al., 2012). In conclusion, there is a current consensus suggesting a reduced leptin signaling in human depression, even though, this association might be stronger in certain subtype of depression or might be influenced by different factors including age, sex, body mass, and co-morbidity with other disorders (Gecici et al., 2005).

Nevertheless, the involvement of the leptin hormone in the emergence of depressive symptoms remains unclear. In this regards, some findings suggest that leptin may exert an antidepressant effect by modulating the hypothalamic-pituitary-adrenal (HPA) axis function. An early *in vitro* study on primary cultures of bovine adrenocortical cells demonstrated the capacity of leptin to reduce the transcription of cortisol (Bornstein and Chrousos, 1999). Mutant mice with altered leptin signaling exhibit hypercorticosteronemia, whereas leptin replacement reduces corticosterone levels in these mice (Chen et al., 1996; Chua et al., 1996; Arvaniti et al., 2001). Similarly, an inverse correlation between basal levels of leptin and release of glucocorticoids (GCs) has been found in humans (Licinio, 1998; Komorowski et al., 2000). Beside a direct effect on GCs, it has also been speculated that leptin may limit the activity of the HPA axis by suppressing the hypothalamic corticotropin-release factor (CRF) release (Ahima et al., 1996; Huang et al., 1998; Arvaniti et al., 2001).

A second hypothesis in favor of the antidepressant role of leptin is linked to the neurotrophic hypothesis of depression, since this adipokine may facilitate neurogenesis. In particular, mutant mice exhibiting an altered leptin signaling present reduced brain volume and abnormal expression of neuronal and glial proteins. These morphological changes have been attributed to the lack of leptin, since leptin administration was shown to normalize the brain morphology (Vannucci et al., 1997; Ahima et al., 1999; Steppan and Swick, 1999). Consistent with a neurotrophic role of leptin, it has been shown that this hormone facilitated the formation of specific neuronal projection pathways inside the hippocampus (Bouret et al., 2004). It also has been shown that leptin increased the motility and density of dendritic filopodia, and enhanced the number of hippocampal synapses (O’Malley et al., 2007). Further, convergent evidence demonstrated the importance in the hippocampus: first, mice with a selectively ablation of Lep-Rb in glutamatergic hippocampal neurons showed long-term potentiation (LTP) impairment, anhedonia-like phenotype, behavioral despair and enhanced social avoidance, and second, specific reduction of leptin receptors in the dentate gyrus, where neurogenesis is considered critical, was shown to induce marked depressive-like behavior (Guo et al., 2013).

In summary, accumulating findings established multiple and complex implications of leptin signaling in physiological and cognitive functions that extend energy balance regulation, and a compromised leptin signaling represents a serious candidate contributing to the development of pathological adaptations underlying the overlap between obesity and depression.

### INFLAMMATION AND CYTOKINES RELEASE

Obesity is associated with a low-grade chronic systemic inflammation and in particularly interleukin-6 (IL-6), TNF-α and C-reactive protein (CRP) are present at high levels in the serum of obese people (Shelton and Miller, 2010; Gregor and Hotamisligil, 2011). Very recent epidemiological studies reported that increased levels of CRP correlated with depressive symptoms and abdominal obesity (Alvarez et al., 2013; Daly, 2013; Wium-Andersen et al., 2013) and other clinical works have found CRP to be the most consistent marker of the obesity-depression association (van Reedt Dortland et al., 2013a,b).

Two factors are responsible for chronic increase of circulating cytokines in obese patients. The first is fat deposition. Fat is stored in the adipocytes of the white adipose tissue and an increased adiposity, especially around the abdomen, stimulate these cells to release inflammatory factors, including adipokines, cytokines, and chemokines (Shelton and Miller, 2010; Gregor and Hotamisligil, 2011). Macrophages attracted into the adipose tissue by chemokines massively produce inflammatory factors, leading to the systemic inflammation observed in human obesity (Clement et al., 1997; Wellen and Hotamisligil, 2003). The second factor is diet quality. In a study conducted on a sample of healthy Greek population, the consumption of several food items was scored during a year. Higher scores, reflecting a stronger adherence to the Mediterranean diet, were inversely associated with biomarkers of systemic inflammation (Chrysohoou et al., 2004). The protective effect of the Mediterranean diet was more recently confirmed by other studies revealing that lower plasma concentration of CRP, IL-6, TNF-α were closely related to this type of diet (Camargo et al., 2012; Rimer et al., 2012; Urpi-Sarda et al., 2012). To date, the identification of the Mediterranean diet nutrients supposed to alleviate signs of chronic inflammation remain unclear. A recent study reviewed 26 randomized clinical trials and established that ω-3 PUFA was of particular interest to lower inflammatory markers (Bloch and Hannestad, 2012; Kiecolt-Glaser et al., 2012; Calder, 2013).

Cytokines are mostly produced in peripheral tissues and due to their large molecular weight cannot freely pass cellular membranes, but they can enter the brain through the leaky regions of the BBB or via cytokine-specific transporters. Cytokine signal also arrives inside the brain by afferent nerve fibers (the vagus nerve for example) or infiltration in the brain parenchyma of peripherally activated monocytes (Plotkin et al., 1996; Rivest et al., 2000; Quan and Banks, 2007; D’Mello et al., 2009). Evidence for elevated levels of neuroinflammatory cytokines has been found in the hypothalamus of rodents after 4 weeks of high-fat feeding, and in *post mortem* brain samples of obese patients as well (Thaler et al., 2012). Noteworthy, whether it is diet-induced or genetically programmed, animal models of obesity present severe inflammation of hypothalamus (Velloso et al., 2008; Wisse and Schwartz, 2009). As a consequence, hypothalamic neurons are injured and the activity of some neurotransmitters, NPY and POMC, compromised (Thaler and Schwartz, 2010; Thaler et al., 2010). Hence, it is likely that excessive amounts of cytokines may exaggerate hypothalamic neuroinflammation and consequently, alter food intake regulation and energy expenditure, worsening signs of obesity in a spiraling down mechanism.

Concomitantly, the neuroinflammation response may exacerbate signs of depression as well, since chronic increase in pro-inflammatory and inflammatory markers has been repetitively observed in depressed patients. It is important to note that inflammation is a protective mechanism for the body to fight microbial and viral attacks. The short-term release of pro-inflammatory cytokines in the blood, including interleukin-1α (IL-1α), interleukin-1β (IL-1β), TNF-α, and IL-6, triggers fatigue, psychomotor retardation, sleep alteration and anhedonia. The “sickness behavior” is supposed to save energy, minimize risks and promote recovery. However, similitudes with the depressive symptoms are quite striking and suggest that depression could be associated with a long-term activity of cytokines (Maes et al., 1995; Charlton, 2000).

In other words, the “cytokine hypothesis of depression” considers depression as the result of a maladaptive response that occurs after a sustained and persistent cytokine release (Dantzer et al., 2008). In favor of this theory, many recent clinical data have shown elevated concentrations of inflammatory markers (including TNF-α, IL-6, IL1-β, and CRP) in the blood and in the cerebral spinal fluid of patient with major depression (Kim et al., 2007; Simon et al., 2008; Howren et al., 2009; Dowlati et al., 2010).

It is also important to note that psychiatric disorders, among which depression, have frequently been described in patient afflicted by pathologies associated with chronic inflammation; it is particularly true for rheumatoid arthritis and autoimmune disorders. Systemic lupus erythmatosus for instance is frequently associated with episodes of major depression. And further, elevated IL-6 spinal fluid levels found in these patients often correlate with the severity of their neuropsychiatric symptoms (Fragoso-Loyo et al., 2007).

The assumption that inflammatory processes may take part in the etiology of depression is also supported by the observation of patients receiving cytokine treatments (e.g., interferon-α) as an anti-cancer or anti-viral therapy. Despite the clinical efficacy of such therapy, more than 45% of treated subjects developed a major depression (Capuron and Miller, 2004; Raison et al., 2006). Moreover, with regards to antidepressant therapies, it has also been noted that their efficacy was related to a reduction of plasmatic levels of inflammatory markers (Lanquillon et al., 2000; O’Brien et al., 2007; Hannestad et al., 2011).

Meanwhile, preclinical findings have demonstrated that mice with targeted deletions of the gene coding for IL-6 or for TNF-α receptor show depressive-resistant phenotypes (Chourbaji et al., 2006; Simen et al., 2006). In other genetically and pharmacologically induced inflammation models, the presence of depressive-like behaviors has been demonstrated in several paradigms including the tail suspension and forced swim tests, as well as in protocols based on social interaction monitoring (Anisman et al., 2005; Moreau et al., 2008; Sukoff Rizzo et al., 2012). Collectively, these findings suggest that depression may represent a maladaptive response to a sustained cytokines release, which may occur in predisposed subjects when the activation of the immune system is heightened in intensity or duration.

While the plasmatic increase of cytokines in depression is confirmed by several studies, little is known about how these modulators of the immune response may affect mood regulation. Recently, it has been proposed that chronic inflammation can affect central neurotransmission, in particular, those of the serotonergic and dopaminergic systems. Particular interest has been accorded to the interaction between cytokines and serotonin because of the serotonergic hypothesis of depression (Coppen and Wood, 1978; Blokland et al., 2002; Wichers and Maes, 2004).

Briefly, cytokines seem to interfere with the serotonin system at different levels: synthesis, release and synaptic reuptake (Dunn and Wang, 1995; Dunn et al., 1999; Anisman et al., 2005). The amount of serotonin in the brain is highly dependent on the availability of the amino acid tryptophan and its transformation in 5-hydroxytryptophan by the enzyme tryptophan hydroxylase (TH; Delgado et al., 1990). Tryptophan levels also depend on an alternative metabolic pathway via its conversion in kynurenine upon the activation of the enzyme indoleamine 2,3-dioxygenase (IDO) localized in multiple cell types including macrophages, astrocytes, and microglia. Hyperactivation of this pathway leads to a depletion of tryptophan and ultimately, decreases the brain amounts of serotonin (Schwarcz and Pellicciari, 2002; Schrocksnadel et al., 2006). Intriguingly, several cytokines (e.g., TNF-α, interferon-α and interferon-γ) and their signaling pathways have been found to activate IDO, hence limiting tryptophan availability (Takikawa et al., 1999; Popov et al., 2006). Consistent with these *in vitro* observations, high plasmatic levels of pro-inflammatory cytokines have been associated with IDO induction in mice displaying depressive-like phenotypes (O’Connor et al., 2009a,b). In line with this interpretation, it also has been shown that: (1) IL-1, IL-6, and TNF-α are able to reduce brain levels of serotonin by accelerating its catabolism (Clement et al., 1997; Dunn, 2006) and (2) cytokines may facilitate serotonin synaptic reuptake (Blakely and Berson, 1992; Bull et al., 2009; Lotrich et al., 2009) hence reducing serotonin neurotransmission.

In parallel, chronic inflammation was shown to alter dopamine neurotransmission as well. Symptoms of depression have been shown to correlate with a reduced prefrontal and striatal dopamine activity (Dunlop and Nemeroff, 2007) and repeated interferon-α administration has been shown to decrease dopaminergic neural activity in the mouse brain (Shuto et al., 1997). Cytokines may also interfere with the dopamine transporter, or affect dopamine synthesis by inhibiting tetrahydrobiopterin (BH4), an important co-factor for tyrosine hydroxylase enzyme that converts tyrosine into L-DOPA (Kitagami et al., 2003). Finally, cytokines also affect the HPA axis functioning. Hyperactivity of the HPA axis is a hallmark of depression (see next section) and several observations have demonstrated that cytokines and cytokine-inducers can potently activate the stress cascade. Administered acutely, cytokines have been shown to increase the expression and the release of CRF, adrenocorticotropic hormone (ACTH), and cortisol (Besedovsky and del Rey, 1996; Pariante and Miller, 2001; Pace and Miller, 2009).

Even though these findings linking cytokines to depression have to be confirmed, the idea that neuroinflammation might be a leading cause of depression, represents a fascinating prospective connecting mood to eating disorders.

## DEPRESSION AS A CAUSE OF OBESITY

The hyperactivation of the HPA axis and reduced neurogenesis and/or brain plasticity are two current hypotheses trying to reconcile the past and recent assumptions about the pathogenesis of depression. Interestingly, these biological processes have been demonstrated to participate to the etiology of obesity as well, and thus may represent a thread union between these two pathologies (Sinha and Jastreboff, 2013; Sominsky and Spencer, 2014).

### HYPERACTIVATION OF THE HPA AXIS

A large body of clinical and preclinical evidence has led to the general consensus that hyperactivation of the HPA axis following a chronic stress experience is a leading cause of depression. Animal and human survival depends on the capacity to recognize and face harmful stimuli (Chrousos, 2009). In this context, stress response allows to react to potentially harmful threat and to maintain body homeostasis through transient physiological and behavioral adaptations (Chrousos and Gold, 1992; McEwen, 1998). This temporary adaptive stress response is directed to increase energy availability in those organs of the body involved to counteract the stressor. As a consequence, cardiac output and respiration are accelerated, catabolism is increased and blood flow is potentiated in the brain and muscles (Gilbey and Spyer, 1993).

These adaptive responses rely on the activation of the autonomic nervous system and the HPA axis, a complex neuro-endocrine system composed of multiple brain structures and peripheral organs (Tsigos and Chrousos, 2002). Emotional and stressful stimuli, processed in the amygdala, activate the paraventricular nucleus of the hypothalamus (PVN) and trigger a cascade of physiological adaptations through the release of corticotropin-releasing hormone/factor (CRH/CRF; de Kloet, 2000; Charmandari et al., 2005). CRF target neurons are located in the anterior pituitary gland and release the ACTH in the bloodstream. This hormone stimulates the cortex of the adrenal gland to secrete GCs, cortisol in humans, and corticosterone in rodents. GCs receptors are widely distributed in the body and their binding with GCs leads to the activation or repression of a plethora of genes (Bamberger et al., 1996; Dostert and Heinzel, 2004), among which those coding for enzymes involved in promoting the hepatic synthesis of glucose from non-glucidic substrates (i.e., lactate, pyruvate, and amino acids). GCs also contribute to increase blood glucose levels, and antagonize the anabolic activities of insulin, growth, and thyroid hormones (Rizza et al., 1982).

When the exposure to stressful stimuli is limited, the stress response has short duration because GCs exert a feedback inhibition on the HPA axis. Specifically, they act on the pituitary and hypothalamus limiting the release of CRF and ACTH, which reduces their own activity. GCs also stimulate GC receptors in the hippocampus, where inhibitory GABAergic projections to PVN neurons block the CRF release (Boudaba et al., 1996; Herman et al., 2002). Higher cortical structures, including the dorsomedial prefrontal cortex and the prelimbic cortex, also control the stress response (Diorio et al., 1993; Radley et al., 2008).

During the past three decades, several groups reported compelling evidence showing that prolonged stress response (in particular sustained CRF and GCs release) may represent a biological mechanism triggering depression (Carroll, 1982; Holsboer, 2000; Pariante, 2003; Strohle and Holsboer, 2003) and obesity (Bjorntorp and Rosmond, 2000; Pasquali and Vicennati, 2000), even though psychosocial stress would exert only a modest effect on weight gain (Wardle et al., 2011). CRF has been particularly studied and its role in the emergence of depressive symptoms has been well documented (Grigoriadis, 2005). The activity of CRF depends on its binding on two types of receptors, both widely express in the brain and body, called CRF-R1 and CRF-R2. Three other endogenous ligands with different affinity for CRF receptors have been revealed and named urocortin1 (UCN1), UCN2, and UCN3 (Nakayama et al., 2011). Consistent with the CRF hypothesis of depression, some studies have shown that depressive patients exhibit high levels of CRF in the cerebrospinal fluid (Nemeroff et al., 1984), increased number of CRF expressing neurons in the PVN, elevated expression of CRF mRNA in the same neurons and reduced CRF receptor density (Banki et al., 1987; Raadsheer et al., 1994; Merali et al., 2004). Early observations in rodents also found that intracerebroventricular (ICV) administration of CRF induced anxiety and depression-like behaviors, whereas the injection of CRF antagonists produced the opposite effect, confirming the potential antidepressive properties of CRF ligands (Deak et al., 1999; Zobel et al., 2000; Seymour et al., 2003). Beside pharmacological data, genetic manipulations in rodents confirmed these observations: overexpression of the CRF gene in mice led to increased anxiety-like behaviors and impaired stress (Stenzel-Poore et al., 1994). Interestingly, these behavioral changes were accompanied by enhanced food intake, weight gain and insulin (Coste et al., 2001). However, whereas transgenic mice lacking CRF-R1 showed a reduced stress response and blunted anxiety-like behaviors (Smith et al., 1998; Timpl et al., 1998), those lacking CRF-R2 exhibited pronounced anxiety-like behaviors and stress hypersensitivity (Bale et al., 2000; Coste et al., 2000).

Compared to depression, less is known about a direct role of CRF signaling on obesity. The involvement of the CRF family peptides (CRF, UCN1, UCN2, and UCN3) in energy balance and food intake regulation has been documented (Chalew et al., 1995; Richard et al., 2002), but most of the data are rather contradictory (Levine et al., 1983; Negri et al., 1985; Asakawa et al., 1999; Ushikai et al., 2011). Similarly to what is observed with anxio-depressive like behaviors, the impact of the CRF system on energy balance largely depends on CRF ligands, the type of receptor and the brain structure targeted. Indeed, recent observations reported that blocking CRF-R1 signaling in the central nucleus of the amygdala prevented palatable food intake and anxiety-like behaviors in the rat (Cottone et al., 2009a; Iemolo et al., 2013). Meanwhile, CRF-R2 activation with UCN2 was shown to reduce high-fat food consumption when injected ICV in the rat, confirming a former observation in mice lacking CRF-R2, which consume larger meals compared to wild type mice (Bale and Vale, 2003; Tabarin et al., 2007).

Further confirming a role of the HPA axis dysfunction in the emergence of excessive food intake and obesity, it has been recently demonstrated in humans that the systemic administration of a low dose of CRF stimulated food intake, likely through GCs release (George et al., 2010). The Cushing’s syndrome, a disease resulting from pituitary/adrenal tumors or chronic treatment with corticosteroids, also represents a coincident model of depression and obesity (Sonino et al., 1998). Clinical observations of these patients revealed that cortisol hypersecretion is accompanied by visceral adiposity, metabolic syndrome and mood related disorders. In order to explain this, early and more recent researches have pointed out that the excessive release of GCs impairs the ability of insulin to promote glucose uptake, induces metabolic syndrome and promotes body fat deposition (Brindley and Rolland, 1989; Black, 2006). Likewise, chronically elevated GCs contribute to visceral fat accumulation in primates (Shively, 1998; Shively et al., 2009) and humans (Marin et al., 1992; Rosmond et al., 1998; Epel et al., 2000; Spencer and Tilbrook, 2011). Finally, the diurnal variations of circulating cortisol levels positively correlated with the hip-to-waist ratio in obese patients (Weaver et al., 1993; Lasikiewicz et al., 2008). Beside insulin and leptin resistance (Zakrzewska et al., 1997, 1999; Jéquier, 2002a,b), GCs may alter the energetic balance and stimulate food intake acting on different brain structures. GCs receptors are widely distributed in hypothalamic nuclei implicated in energy homeostasis such as ARC, LH, and PVN (Morimoto et al., 1996) and long-lasting stimulation of GC receptors in these brain regions is known to potentiate orexigenic signals modulating the expression of genes involved in energy balance. In particular, the expression of one gene contributing to satiety signaling, the POMC gene, has been found to be reduced following GC receptor stimulation and increased after adrenalectomy (Cavagnini et al., 2000; Savontaus et al., 2002).

While clinical data strongly support that chronically elevated GCs receptors affect the hedonic regulation of food intake and increase the preference for palatable food, which most likely contributes to excessive fat deposition (Dallman et al., 2003, 2006; Pecoraro et al., 2004; Germano et al., 2007), enhanced levels of corticosterone have been found in different animal models of obesity including fa/fa rats, db/db, and ob/ob mice (Guillaume-Gentil et al., 1990). Conversely, the absence of GCs, due to adrenalectomy, provoked body weight loss and food intake reduction in rodents, effects that could be reversed by corticosterone administration (Saito and Bray, 1984; Pralong et al., 1993; Makimura et al., 2000). Accumulating evidence suggests that diet composition may also play a role in modulating the HPA axis functions. Although, only a few studies have covered this topic, some of them indicate that unbalanced fat diets may perturb lipid metabolism and, as a consequence, increase circulating levels of GCs. Consistent with this hypothesis, a recent study on woman health has reported a positive correlation between consumption of saturated fatty acids and diurnal variation of cortisol (Garcia-Prieto et al., 2007). In addition, converging evidence has found that patients who received diets supplemented with PUFA or fish oil had reduced ACTH and cortisol rise after acute stress (Delarue et al., 2003; Michaeli et al., 2007).

Collectively, these observations emphasizes that stress alters the HPA axis homeostasis and most likely triggers the onset and worsening of depression. Since the hypothalamus is the main brain orchestrator regulating energy balance and food behavior, it is not surprising that alterations in the HPA axis may lead to overeating and obesity.

### NEUROGENESIS AND THE BRAIN-DERIVED NEUROTROPHIC FACTOR (BDNF)

The monoaminergic hypothesis of depression has long proposed that depression originates from decreased monoamine neurotransmission. Despite the impressive number of pre-clinical and clinical studies that have been conducted over the last 40 years, the validity of this theory remains debatable (Mulinari, 2012; Blier and El Mansari, 2013). In particularly, the discrepancy between the rapid increase of monoamine signaling occurring upon antidepressant treatment and their delayed therapeutic effect remains unclear. Moreover, inconsistent clinical findings have failed to demonstrate a decrease in monoamine basal levels in depressed patients compared with health people. Thus, the altered monoamine neurotransmission classically reported in depression is now considered to reflect the consequences of neuronal damage. Historically neurons have been presumed to not regenerate. However, recent evidence demonstrated that the mammalian central nervous system retains the capacity to produce new neurons that can integrate neural network (Ming and Song, 2011). Two brain regions have been extensively studied: the subventricular zone of the lateral ventricles and the subgranular region of the dentate gyrus in the hippocampus (Duman et al., 2001; Duman, 2002; Kempermann and Kronenberg, 2003). Hippocampal neurogenesis, which is pivotal for cognitive functions and mood control, is strongly affected by chronic stress which causes shortening and pruning of neuron dendrites and reduction of the final number of newborn neurons (McEwen, 1999; Duman, 2004a,b). Accordingly, morphologic and morphometric studies of brains of depressed patients have found a reduction of hippocampal volume due, at least in part, to an impaired neurogenesis (Sheline, 1996; Bremner et al., 2000; Sheline et al., 2003; Stockmeier et al., 2004; Lucassen et al., 2010). Interestingly, antidepressant treatments have shown to reverse neural atrophy, cell loss and stimulate neurogenesis (Duman and Monteggia, 2006). Similar observations have been reported with preclinical investigations, confirming that unpredictable chronic stress protocols suppressed hippocampal neurogenesis in rodents (Watanabe et al., 1992; Malberg and Duman, 2003; Pham et al., 2003; Mineur et al., 2007).

Neurotrophins constitute an important class of signaling molecules in the brain, playing a pivotal role in brain development, neuron survival and synaptic plasticity. The brain-derived neurotrophic factor (BDNF) has received particular attention, since it is considered a relevant biomarker of depression and suicidal behavior, and a possible downstream target of a variety of antidepressant drugs (Dwivedi, 2009; Lee and Kim, 2010). Hippocampal neurogenesis is an important process that appears to be involved in maintaining balanced mood. A large body of evidence identifies BDNF, and its TrkB receptor, as two key elements in the orchestration of different phases of neurogenesis such as cell proliferation, migration, differentiation and death. The rationale for the involvement of BDNF in depression comes from the observation that stress can down-regulate the expression of this neurotrophic factor in brain structures known to control emotions (Dwivedi, 2009; Lee and Kim, 2010). Stress-related disorders, among which major depression, is considered to induce morphologic damages in the hippocampus, which is intimately connected to the HPA axis (Bremner et al., 2000). Accordingly, the expression of BDNF, BDNF regulated genes and TrkB receptors are decreased in *post mortem* hippocampal tissues harvested from depressed human brains (Tripp et al., 2012). Similar findings have also been reported for the prefrontal cortex, another brain region essential for mood regulation. Moreover, BDNF protein levels are reduced in the serum of depressed patients (Castren et al., 2007; Castren and Rantamaki, 2010; Thompson Ray et al., 2011). It is important to note that, *post mortem* hippocampal BDNF levels and serum concentrations were normalized in depressed patients successfully treated with antidepressants, suggesting that the therapeutic effect of these compounds is related to BDNF activity (Duman and Monteggia, 2006).

However, human studies are only correlative and a clear causal association between impairment of BDNF brain activity and depression has not been yet demonstrated in human patients. Thus, preclinical studies have attempted to validate this theory. Converging studies have shown that stress (both physical and psychological) is able to lower BDNF expression levels in rat hippocampus (Duman and Monteggia, 2006) and that direct BDNF infusion in this area reduces stress-induced depressive-like behaviors (Hoshaw et al., 2005; Hu and Russek, 2008). Viral-mediated deletion of BDNF or TrkB genes in the VTA resulted in a significant antidepressant-like response as well. However, results discrepancy made complicated the interpretation. For instance, mice exposed to a chronic social defeat exhibited increased depressive-like behaviors and increased BDNF protein levels in the nucleus accumbens and amygdala (Berton and Nestler, 2006; Yu and Chen, 2011). Therefore, the current hypothesis, suggesting a strong link between low BDNF brain levels and depression, appears to be too simplistic and has to be reconsidered. One possible explication is that BDNF gene expression may be down regulated in some brain structures (like hippocampus and prefrontal cortex) and up regulated in others.

Interestingly, compelling evidence demonstrated that BDNF has a direct role in the regulation of homeostatic and hedonic eating as well (Lyons et al., 1999; Kernie et al., 2000; Rios et al., 2001; Xu et al., 2003). With regards to the homeostatic regulation, early studies showed that ICV injection of BDNF in rats led to a reduction of body weight, suggesting that BDNF could take part in the central control of feeding behavior (Lapchak and Hefti, 1992; Pelleymounter et al., 1995b). More recently, BDNF deficiency has been associated with increased weight in mice (Noble et al., 2011; Schwartz and Mobbs, 2012), while leptin injected in the ventromedian hypothalamus (VMH) would exert an anorexigenic effect activating the expression of BDNF (Komori et al., 2006). The relevance of BDNF in energy balance was also confirmed in mutant mice. Heterozygous BDNF +/- mice show hyperphagia, body weight gain, insulin resistance, dyslipidemia, and hyperglycemia. In addition, these mice were more sensitive to negative effects of a high fat diet (Lyons et al., 1999; Kernie et al., 2000). Similarly, mice expressing only about 25% of TrkB receptors display excessive feeding (Xu et al., 2003). Inversely, BDNF infusion in the VMH of adult wild-type mice resulted in decreased food intake and body weight (Wang et al., 2007). Consistent with a role in homeostatic mechanisms, levels of expression of BDNF and trkB in hypothalamus and hindbrain regions are influenced by the energy status (Xu et al., 2003; Bariohay et al., 2005; Tran et al., 2006; Unger et al., 2007). Preclinical studies also support a role for BDNF in regulating hedonic feeding by modulating the mesolimbic dopamine (Seroogy et al., 1994; Numan and Seroogy, 1999; Cordeira et al., 2010). Meanwhile, only a limited number of human studies managed to correlate BDNF expression to obesity. Human BDNF haploinsufficiency was linked to elevated food intake and obesity (Gray et al., 2006; Han et al., 2008). Recent evidence also associated a functional polymorphism of the Bdnf gene, Bdnf Val66met which impedes a correct secretion and signaling of BDNF, with obesity predisposition (Beckers et al., 2008; Skledar et al., 2012), and the missense mutation in the TrkB gene, which prevents TrkB function, has been identified in patients exhibiting overweight and severe obesity (Yeo et al., 2004).

Interestingly, adhering to a balanced diet was shown to influence neurogenic factors, and potentially alleviates signs of depression and obesity. Depressed patients following a Mediterranean diet exhibited signs of remission concomitant to increased plasma BDNF concentrations (Sanchez-Villegas et al., 2011; Sanchez-Villegas and Martinez-Gonzalez, 2013). Meanwhile, overweight and obese patients exposed to a 3-months calorie restricted diet displayed increased levels of serum BDNF (Araya et al., 2008). On the other hand, high fat meals were shown to decrease plasma BDNF of almost 30% in healthy patients (Karczewska-Kupczewska et al., 2012). These observations have been confirmed in preclinical studies since BDNF level decreased in rats maintained on a high carbohydrate diet (Maioli et al., 2012), and high fat diet (Yamada-Goto et al., 2012), whereas caloric restriction increases BDNF expression (Lee et al., 2002; Duan et al., 2003).

Further investigation has revealed that ω-3 PUFA may play a role in neurogenesis. In animals, ω-3 PUFA supplementation provided protection against reduced plasticity and normalized BDNF after traumatic brain injury (Wu et al., 2004), whereas diets deficient in ω-3 PUFA lowered BDNF brain levels (Rao et al., 2007; Bhatia et al., 2011).

Taken together these data suggest that BDNF pathway may be a relevant biological substrate underlying the pathogenesis of both mood and eating disorders. However, considering the emerging data on complexity of BDNF pathway, further findings are needed to better understand whether BDNF is a real causal factor for the depression-obesity association.

## CONCLUDING REMARKS

Obesity and depression represent a global health burden, and the diagnostic is even more severe for those individuals suffering from both diseases. The increasing prevalence of depression-obesity co-morbidity strongly suggests that these disorders may share a common pathogenesis.

Earlier and current findings clearly demonstrated a link between the two pathologies but the nature of their association is still to be fully understood. The complexity comes from the fact that both obesity and depression are heterogenic diseases, influenced by multiple environmental and genetic factors. Clinical works have identified environmental factors that seem to facilitate the development of obesity as well as that of depression. In particular, factors like stress and diet quality have a strong impact on both pathologies and may constitute key mediators for the obesity-depression association. Other risk factors, reduced physical activity, sleep impairments and altered circadian rhythms, are also considered relevant but most likely as factors worsening the acquired pathologies rather that factors influencing the emergence of the diseases.

Collectively, clinical studies reviewed in this article suggest that obesity and depression are closely related but may not be globally interconnected. Indeed, only subgroups of obese patients are at higher risk for developing depression, and *vice versa*. Accumulating evidence emphasizes that mainly patients with BEDs and those with abdominal fat deposition and metabolic syndrome are at higher risk for depression. The identification of these two subgroups of obese individuals is promising for discovering the biological mechanisms underlying the obesity-depression association.

In the last years, clinical and preclinical studies have also found that leptin may represent a biological substrate underlying the pathogenesis of both obesity and depression. Circulating leptin increases proportionally with body mass and is significantly elevated in obese patient in comparison with non-obese individuals. However, persistent high levels of the hormone alter leptin signaling in the brain, most likely due to leptin resistance compensatory mechanisms. This deficit is believed to trigger a maladaptive functioning of the brain homeostatic system, ultimately leading to obesity. Since converging evidence suggests that impaired leptin signaling may affect mood regulation and food reward perception in humans, impaired leptin signaling cascades may represent a biological mechanism binding obesity and depression, in particular when obesity is paired with compulsive overeating. This assumption opens novel perspectives for the development of therapeutic medications able to correct clinical signs of obesity and depression.

Substantial fat deposition is known to stimulate inflammatory processes, which in turn, promote peripheral inflammatory cytokines release. These molecules enter the brain, not only affecting the hypothalamic control of food intake but also impacting other brain functions that are pivotal for mood regulation. Consistent with this hypothesis, some findings demonstrated that high levels of inflammatory cytokines might interfere with serotonin and dopamine neurotransmission, and with HPA axis as well. Although they need to be confirmed, these findings suggest that the inflammatory hypothesis of eating and mood disorders should get a larger attention in the near future.

The stress response is known to alter the HPA axis functioning and to trigger depressive symptoms and eating disorders. Deciphering the contribution of CRF signaling in these two pathologies remains quite difficult though. Indeed, CRF has opposite effects on depressive symptoms depending on which brain receptors are activated, but CRF binding to CRF receptor 1 seems to exacerbate signs of depression while increasing food intake. The development of CRF receptor 1 antagonists may represent another relevant strategy for improving mood and eating disorders. Inhibitors of GCs are currently used for treating the Cushing’s syndrome and some cancers of the adrenal gland, but no medications are available to date for treating depression or obesity.

The neurothrophic hypothesis of depression has received particular attention, but the current knowledge remains elusive and recent evidence, rather than establishing a clear-cut role for BDNF to causally link depression to obesity, mainly claim for further studies before delineating any mechanism underlying the association obesity-depression.

In conclusion, compelling evidence shows that obesity and depression are two overlapping pathologies. However, the causal link between eating and mood disorders needs to be clarified. Most likely, different mechanisms may contribute to the worsening of each disease, and a global cure does not sound realistic. Instead, a better understanding of the molecular and cellular adaptations occurring in subgroups of obese (or depressed) patients identified as highly vulnerable to develop comorbidities should be a clinical priority. Meanwhile, improving animal models of eating and mood disorders is critical for unraveling the underpinnings of the obesity-depression association.

However, given the deleterious impact of stress and junk food consumption on the onset and progression of these two pathologies, the most effective prevention program remains public campaign defending the adherence to healthy balanced diets and promoting effective programs of stress management.

## Conflict of Interest Statement

The authors declare that the research was conducted in the absence of any commercial or financial relationships that could be construed as a potential conflict of interest.
